
JOURNAL INFORMATION
==============================
NLM Title Abbreviation: Alcohol Res Health
Journal ID: Alcohol Res Health
NLM Journal ID: 100900708
Journal ID: ARH

Alcohol Research & Health

ISSN: 1535-7414
EISSN: 1930-0573
Publisher: National Institute on Alcohol Abuse and Alcoholism

ARTICLE INFORMATION
==============================
PMCID: PMC3860442
Article ID: arh-31-1-84
Article version: 1
Subjects: Glossary

Glossary

Print publication date: 2008
Volume: 31
Issue: 1
First page: 84
Last page: 86
Copyright year: 2008
License: Unless otherwise noted in the text, all material appearing in this journal is in the public domain and may be reproduced without permission. Citation of the source is appreciated.
License URL: https://creativecommons.org/publicdomain/mark/1.0/

Comment in: Commentary: Systems Biology and Its Relevance to Alcohol Research 31 1 2008 5 10 Alcohol Research & Health PMC3860440 23584746
Comment in: Systems Genetics of Alcoholism 31 1 2008 14 25 Alcohol Research & Health PMC3860445 23584748
Comment in: Proteomic Approaches for Studying Alcoholism and Alcohol-Induced Organ Damage 31 1 2008 36 48 Alcohol Research & Health PMC3860448 23584750
Comment in: Mathematical Modeling of Complex Biological Systems 31 1 2008 49 59 Alcohol Research & Health PMC3860444 23584751
Comment in: Metabolomics in Alcohol Research and Drug Development 31 1 2008 27 35 Alcohol Research & Health PMC3860450 23584749

==============================
Allele One of two or more variants of a certain gene.

Amino acids Building blocks of proteins, characterized by the presence of an amino group (NH2), a carboxy group (COOH), and a side chain that differs for each amino acid; proteins are defined by their sequence of amino acids.

Antibodies Immune molecules naturally produced by the body that recognize foreign or harmful molecules that have entered the body, bind to these molecules, and mark them for destruction by the body’s immune system. Antibodies that specifically interact with a given protein also can be produced in laboratory animals, such as mice, chickens, or rabbits.

Apoptosis Series of biochemical reactions occurring in a cell whereby cells that are damaged or which are no longer needed undergo a process of self-destruction; also known as programmed cell death or cell suicide.

Bioinformatics The science of managing and analyzing biological data using advanced computing techniques.

Cell cycle The series of events that take place in a cell leading to its replication, including a growth period during which the cell grows, a division phase during which the cell splits itself into two distinct daughter cells, and a resting phase between the division phase and the next growth period.

Cerebellum The region of the brain that controls sensory perception and motor functions.

Chaperone Protein that assists in the folding/unfolding and in the assembly/disassembly of other large molecules in the cell (e.g., other proteins); one major function of chaperones is to prevent amino acid chains synthesized during translation to fold into a nonfunctional three-dimensional shape.

Chromatography Method to separate mixtures of molecules (e.g., proteins) based on their differential movement through a two-phase system (e.g., a solid support material and a liquid buffer). The movement of each molecule through the two phases is determined by various factors (e.g., size, electric charge, interactions with components of the support material).

Chromosome The organized form of DNA found in cells that contains many genes, regulatory elements, and other intervening nucleotide sequences as well as proteins that help give chromosomes their compact structures.

Citric acid cycle A series of enzyme-catalyzed chemical reactions involved in the metabolism of glucose (and other metabolic pathways) that plays a role in the cell’s energy production, in the synthesis of several nerve cell signaling molecules (neurotransmitters), and in protein synthesis.

Continuous model Mathematical model that treats a reaction as occurring continuously, not only at specific time points or intervals.

Cytoskeleton The cellular “scaffolding” contained within cells that helps the cells maintain their shape, enables cell movement, and is important for transport of cell components within the cell as well as for cell division. The fibers of the cytoskeleton consist of various proteins (e.g., actin).

Differential gene splicing Biochemical process that occurs after transcription and which involves excising specific sections of the initial transcription products, resulting in diverse mature messenger RNA (mRNA) molecules that can be translated into different proteins.

Differentiation Process whereby newly formed embryonal cells assume their specialized characteristics and functions.

Electrophoresis Method for separating mixtures of biomolecules (e.g., proteins, DNA) as they move through a support material exposed to an electric field. The distance each molecule travels depends on its size and electrical charge.

Emergent properties The characteristics of a complex system that arise from the interaction of various components.

Endophenotype Hereditary characteristics that normally are associated with certain conditions but are not direct symptoms of that condition.

Epistasis The interaction among genes.

Eukaryotic cell Cell of a higher organism that is characterized by the presence of a cell nucleus.

Fatty acid A major component of fats that is used by the body for energy and tissue development.

Feedback loops Regulatory mechanisms by which the end products of a certain process help to shut off or enhance further initiation of that process.

Flux Amount that flows through a unit area per unit time.

Gene expression The conversion of the genetic information encoded in the DNA into proteins.

Genome The entirety of all genes of an organism.

Genomics The study of the structure and function of an organism’s complete genetic content, or genome.

Genotype The complete genetic makeup of an organism determined by the particular combination of alleles for all genes.

Glycolysis Initial set of biochemical reactions in the metabolism of glucose, during which glucose is converted to either lactate or pyruvate (which can enter the citric acid cycle).

Haplotype A set of closely linked genetic markers present on one chromosome, which tends to be inherited together (not easily separable by recombination). Some haplotypes may be in linkage disequilibrium.

Hexokinase An enzyme involved in glycolysis.

Huntington’s Disease A genetically programmed degeneration of nerve cells (i.e., neurons), in certain areas of the brain, causing uncontrolled movements, loss of intellectual faculties, and emotional disturbance.

Insulin resistance Condition in which normal amounts of insulin are inadequate to produce a normal insulin response from fat, muscle, and liver cells; results in elevation of free fatty acids and glucose levels in the blood plasma; often leads to a condition called metabolic syndrome and type 2 diabetes.

Interaction proteomics Proteomic approaches focusing on identifying proteins that interact to form large functional complexes or even networks of proteins involved in regulating one specific process.

Irreducible system A complex system that cannot be fully understood by studying its individual components by themselves because of the system’s emergent properties. Complex diseases such as alcoholism, which are caused by multiple genes and other factors, also can be considered irreducible systems because study of the involved genes and environmental factors by themselves likely cannot explain all aspects of the disease.

Ischemia A condition in which blood flow is restricted to a part of the body.

Isoelectric point (pI) The pH at which a particular molecule (e.g., a protein) carries no net electrical charge. The pH represents the acidity or alkalinity of a solution and is determined by the number of free protons (H+) in the solution. The higher the concentration of H+, the lower the pH. At a pH below the pI, proteins carry a net positive charge; above the pI they carry a net negative charge. The net electrical charge of a protein determines how far it migrates during electrophoresis.

Isotope Any of the several different forms of a chemical element that differ in their atomic mass (mass number); isotopes of an element possesses the same number of protons in their nuclei but different numbers of neutrons and therefore differ in their mass numbers, which reflect the total number of protons plus neutrons.

Kinase An enzyme that transfers phosphate groups from one molecule (the donor) to a specific target molecule (the substrate).

Kinetic properties Properties of a chemical reaction or of an enzyme mediating a reaction that determine how fast this reaction occurs (e.g., the rate at which the starting material [substrate] is converted into the reaction product or the strength with which an involved enzyme binds to its substrate).

Linkage disequilibrium Occurrence of some genes together more often than would be expected.

Locus/loci The position a gene occupies in a chromosome.

Macromolecule A very large molecule, such as DNA or a protein, consisting of many smaller structural units linked together.

Mass spectrometry (MS) Method to identify proteins isolated by electrophoresis or chromatography. For MS, the isolated proteins are cut into peptides by enzymes called proteases, and the sizes of the resulting peptides are determined through one of several approaches. Based on the measured masses of all peptides, computers can determine the most likely protein from which they were derived.

messenger RNA (mRNA) Key intermediary molecules that are generated when a gene is expressed (i.e., the information encoded in the gene is converted into a protein product by the cell).

Metabolic flux The rate of flow of a metabolite (or a certain chemical element, such as carbon or nitrogen) along one or more metabolic pathways or through a single enzyme reaction.

Metabolism The totality of chemical reactions occurring in a cell, an organ, or the body. The term sometimes is applied more narrowly to the breakdown of a particular substance by specific enzymes.

Metabolite Intermediary product generated during the metabolism of a particular molecule; the levels of these metabolites within a cell, tissue, or an organism largely reflect the endogenous activity of biochemical pathways but also are responsive to external or environmental changes.

Metabolome The entirety of all metabolites present in a given cell, tissue, or organism.

Microarray A chip made from glass, plastic, or other type of support material onto which a large number of minute samples (e.g., proteins, DNA samples) are affixed in an orderly manner for performing automated assays.

Microsomes Small bodies in the cell where certain substances are metabolized.

Mitochondria Structures within cells that generate most of the cells’ energy through the production of adenosine triphosphate, a molecule that provides the energy needed for many key metabolic reactions.

Multiscale model Mathematical model that can incorporate effects occurring on very different length or time scales (e.g., effects occurring within seconds as well as effects occurring within days).

Neocortex Outermost layer and most prominent structure of the mammalian brain; involved in higher cognitive functions, such as thought, language, and behavior.

Neurogenesis Formation of nerve cells (neurons) during embryonal development.

Nucleic acid A macromolecule composed of nucleotide chains. Nucleic acids carry genetic information or form structures within cells.

Nucleotide A subunit of DNA and RNA consisting of a nitrogen-containing base, a phosphate group, and a sugar molecule.

Oxidation A chemical reaction that results in a loss of electrons, which are negatively charged subatomic particles, by a substance and which usually involves removing a hydrogen atom from a molecule or adding oxygen to it, or both.

Pentose phosphate pathway A metabolic process that serves to generate NADPH (a cofactor required for many enzyme reactions) and 5-carbon (i.e., pentose) sugars from glucose. It consists of two distinct phases: an oxidative phase, during which NADPH is generated, and a nonoxidative phase during which the 5-carbon sugars are formed. This pathway is an alternative to glycolysis.

Peptide A short chain of amino acids as opposed to a protein, which is defined as a long chain of amino acids; proteins can be cut into peptides by proteases.

(Lipid) Peroxidation The process whereby free radicals “steal” electrons from the lipids in the cell membranes, resulting in cell damage and increased production of free radicals.

Phenotype The observable properties, traits, or physical appearance of an organism resulting from the interaction of the genotype with environmental factors.

Plasma The yellow-colored liquid component of blood in which blood cells are suspended.

Polymorphism Existence of a gene in several allelic forms.

Posttranslational modifications (PTMs) Modification of newly synthesized proteins by the addition of various chemical groups (e.g., phosphate groups or sugar molecules); these PTMs can make a protein more or less active.

Proliferation Growth and increase in numbers of cells; achieved by the division of cells into daughter cells.

Protease An enzyme that cleaves proteins into smaller pieces (i.e., peptides). Many proteases (e.g., trypsin) cleave the proteins only at specific sites characterized by a specific sequence of amino acids in the protein. Consequently, each protein yields a specific set of peptides after trypsin treatment.

Proteome The entirety of all proteins of a cell, tissue, or organism.

Proteomics The large-scale study of the structure and functions of proteins.

Quasi-stationary Not changing significantly over time; for example, if a chemical reaction is quasi-stationary, generally the same amount of starting material enters the reaction over a given period of time as leaves the reaction in the form of the reaction products.

Shotgun proteomics Proteomic approaches focusing on separating and identifying all proteins in a given cell or tissue, without any prior selection of specific groups of proteins or knowledge of the proteins’ functions.

Single nucleotide polymorphism (SNP) Genetic variation that results from the exchange of only a single nucleotide.

Stochastic Random, unpredictable; a stochastic process is one in which one state does not fully determine the next state.

Stoichiometric Related to the determination of the relative quantities of the different substances involved in a chemical reaction (e.g., if twice as many hydrogen atoms as carbon atoms are present in the starting substances of a chemical reaction, twice as many hydrogens as carbons must also leave the reaction in the reaction products).

Synapse Region of a nerve cell (neuron) from which nerve signals are transmitted to neighboring neurons.

Synaptogenesis Formation of synapses between neurons during brain development.

Thiamine Vitamin B1; essential cofactor of several enzymes involved in the metabolism of glucose.

Transcription Biochemical process in which an intermediary molecule called messenger RNA (mRNA) is generated based on the genetic information of the DNA.

Transcription factor Protein regulating gene expression; consists of at least two functional domains: a DNA-binding domain and an activating domain.

Transcriptome The entirety of all transcription products (transcripts or messenger RNA [mRNA]) present in a cell, tissue, or organism.

Transcriptomics The large-scale study of the expression level of messenger RNA (mRNA) in a given cell, organ, or organism.

Transferrin A protein found in the blood that helps deliver iron to the cells (e.g., to the developing red blood cells that need iron to transport oxygen).

Translation Biochemical process during which messenger RNA (mRNA) serves as a blueprint based on which proteins are synthesized from their building blocks, the amino acids.

Triglycerides The chemical form in which most fat molecules exist in food as well as in the body; they consist of glycerol and three fatty acids. Triglycerides also are present in blood plasma and, together with cholesterol, form the plasma lipids.

